# A personalized DVH prediction model for HDR brachytherapy in cervical cancer treatment

**DOI:** 10.3389/fonc.2022.967436

**Published:** 2022-08-30

**Authors:** Zhen Li, Kehui Chen, Zhenyu Yang, Qingyuan Zhu, Xiaojing Yang, Zhaobin Li, Jie Fu

**Affiliations:** ^1^ Shanghai Jiao Tong University Affiliated Sixth People's Hospital, Shanghai, China; ^2^ Shuguang Hospital Affiliated to Shanghai University of Traditional Chinese Medicine, Shanghai, China; ^3^ Duke University, Durham, NC, United States

**Keywords:** machine learning, brachytherapy, cervical cancer, dose prediction, radiation oncology

## Abstract

**Purpose:**

Although the knowledge-based dose-volume histogram (DVH) prediction has been largely researched and applied in External Beam Radiation Therapy, it is still less investigated in the domain of brachytherapy. The purpose of this study is to develop a reliable DVH prediction method for high-dose-rate brachytherapy plans.

**Method:**

A DVH prediction workflow combining kernel density estimation (KDE), k-nearest neighbor (kNN), and principal component analysis (PCA) was proposed. PCA and kNN were first employed together to select similar patients based on principal component directions. 79 cervical cancer patients with different applicators inserted was included in this study. The KDE model was built based on the relationship between distance-to-target (DTH) and the dose in selected cases, which can be subsequently used to estimate the dose probability distribution in the validation set. Model performance of bladder and rectum was quantified by |ΔD_2cc_|, |ΔD_1cc_|, |ΔD_0.1cc_|, |ΔD_max_|, and |ΔD_mean_| in the form of mean and standard deviation. The model performance between KDE only and the combination of kNN, PCA, and KDE was compared.

**Result:**

20, 30 patients were selected for rectum and bladder based on KNN and PCA, respectively. The absolute residual between the actual plans and the predicted plans were 0.38 ± 0.29, 0.4 ± 0.32, 0.43 ± 0.36, 0.97 ± 0.66, and 0.13 ± 0.99 for |ΔD_2cc_|, |ΔD_1cc_|, |ΔD_0.1cc_|, |ΔD_max_|, and |ΔD_mean_| in the bladder, respectively. For rectum, the corresponding results were 0.34 ± 0.27, 0.38 ± 0.33, 0.63 ± 0.57, 1.41 ± 0.99 and 0.23 ± 0.17, respectively. The combination of kNN, PCA, and KDE showed a significantly better prediction performance than KDE only, with an improvement of 30.3% for the bladder and 33.3% for the rectum.

**Conclusion:**

In this study, a knowledge-based machine learning model was proposed and verified to accurately predict the DVH for new patients. This model is proved to be effective in our testing group in the workflow of HDR brachytherapy.

## 1 Introduction

Cervical cancer is the fourth most common cancer in women globally (1). The treatment of cervical cancer relies on a combination of external radiotherapy and HDR brachytherapy to increase the dose being delivered to the primary tumor (2, 3). Numerous studies have shown that high-dose-rate brachytherapy (HDR-BT) is strongly correlated with patients’ survival rates and plays an essential curative role in cervical cancer (4, 5). It allows delivery of highly localized doses to the target and excellent sparing of surrounding organs at risk (OARs). High-quality planning is a critical component in gynecologic BT treatment. However, unlike in external-beam radiation therapy (EBRT), the planning workflow in BT necessitates the collaboration of multidisciplinary teamwork in the shortest possible time to minimize the patient’s discomfort and movement. The pressures of a fast-paced and accurate planning will put the entire workflow under stress, raising the risk of planning errors. Additionally, the experience and preference of brachytherapy planners, as well as clinical expertise and intuition would result in large inter- and intra- plan quality variations, further introducing more uncertainties to the BT treatment.

In the last decade, Knowledge-based planning (KBP), a new set of data-driven methodologies has been developed to improve the quality and efficiency of EBRT planning based on the previous high-quality clinical plans (6–9). Many researchers have demonstrated KBP’s strength and validity in guiding planners to achieve optimal dose-volume histograms (DVHs) for OARs in treatment planning (10–12). KBP methods in EBRT are usually classified into two major categories: (a) case and atlas-based methods; and (b) statistical modeling and machine learning methods. In general, case and atlas-based methods utilize geometric features to find the best-matched prior cases from a database to improve the current planning. The statistical modeling and machine learning approaches try to build dose prediction models based on regression models such as stepwise regression (13, 14), multivariate linear regression (15–17), support vector regression (18, 19), and logistic regression (20, 21). After years of development, KBP methods have been widely investigated and even clinically implemented in commercial treatment planning systems. RapidPlan™, a commercial software module integrated into Eclipse released by Varian (Varian Medical Systems, Palo Alto, CA, USA), is one such system that predicts the achievable doses and specifies the optimization objectives needed to achieve them based on the KBP method. The success of RapidPlan™ has demonstrated the KBP’s practicability and effectiveness in EBRT (22, 23).

All these achievements verified the success KBP achieved in model accuracy, stability, and feasibility in clinical application in the area of EBRT. As mentioned above, the unique clinical workflow in BT makes it vulnerable to suboptimal plans. Quality control tools such as KBP are especially important, and promising in detecting suboptimal plans in BT. Moreover, current recommendations and guidelines have provided various dose constraints for BT planning. These population-based guides mainly concentrate on ensuring normal tissues do not exceed the dose limits, rather than guaranteeing individual patient receive the optimal dose distribution for their anatomy (24). Therefore, patient anatomy-based KBP, which could be used as patient-specific dose predictions to quantify currently unknown quality variations, would be particularly useful for brachytherapy.

However, despite great success achieved in EBRT, KBP is still far relatively unexplored in HDR brachytherapy due to some exceptional challenges in brachytherapy. The main reason is that the dose distribution in BT plans is highly constrained around the inserted applicator, resulting in insufficient freedom of dose modulation in planning. To the best of our knowledge, only two studies have investigated the possible application of KBP in brachytherapy, and both focused on tandem and ovoid applicator in an intracavitary setting. In Yusufaly et al.’s study (25), the authors used an established external-beam knowledge-based DVH estimation method to predict D_2cc_ in OARs. Zhou et al. (26) proposed a support vector machine model for dose prediction and effectively predicted D_2cc_. Both studies demonstrated good results in predicting critical brachytherapy dose metrics using intracavitary brachytherapy applicators. Currently, only Tandem and Ovoid applicators were investigated in the knowledge-based planning strategies. However, various applicators with different geometry and source position would increase the dose distribution’s versatility and complexity.

In this study, we proposed a KBP method to estimate the OAR dose distribution in HDR-BT treatment. To our knowledge, this work is the first application of knowledge-based dose estimation in HDR-BT with both intracavitary and interstitial cases included.

## 2 Method

### 2.1 Prediction pipeline

**Figure 1:**
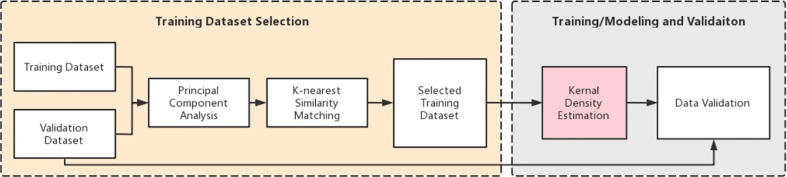
Pipeline of the DVH prediction.

The study consists of two main tasks: (a) training dataset selection and (b) model training and validation. **Figure 1** shows an overview of the entire workflow. Briefly, we first performed the principal component analysis (PCA) for all the training cases. Then, we retrieved the top k (bladder: k=30, rectum: k=20) plans from the training database based on PCA results using the k-nearest neighbor (kNN) algorithm. Cases in the new dataset have the most similar features to the cases in the validation dataset. These selected cases are then used to create a new training dataset to build training models. Subsequently, we used kernel density estimation (KDE) to develop a robust prediction model based on the selected k plans. After training, a series of validation tests were conducted.

### 2.2 Patient database

79 HDR cervical patients previously treated in our center from 2019 to 2021 were included in this retrospective study. Each patient consists 2-4 fraction, with a total of 216 cases/fractions were involved. In this study, a ‘case’ indicates one single fraction containing a unique simulation CT and a treatment plan. All the cases were randomly divided into two datasets, with 170 cases in the training dataset and 46 cases in the validation dataset.

OARs and HRCTV were contoured based on CT images acquired at a GE 128 slice CT (Discovery, GE Healthcare, Inc.) using a tube setting of 120kV and 60mAs. All of the scans used identical parameters for image acquisition and reconstruction. The slice thickness and slice increment were 2.5*2.5mm. Each patient was treated with an applicator set among Tandem and Ovoid applicator (T+O), Ovoid applicator alone, Vaginal Multi-Channel applicator, 3D printed applicators, free needles, and a tandem applicator with up to 10 interstitial needles (T+N). The overview of the treatment characteristics is shown in **Table 1**.

**Table 1:** Treatment characteristics of included patients.

	Training Dataset	Test Dataset
**Applicator Type**		
Tandem + Needles	26	8
Vaginal	34	8
Tandem + Ovoid	45	15
Ovoid	28	5
Free Needles	26	6
3D_Printed	11	4
Total	170	46
**Prescription Dose**		
4	10	5
4.5	5	2
5	40	12
5.5	15	3
6	100	24
Total	170	46

Prescription dose and applicator types are grouped based on data from all included fractions(cases) per patient.

According to the OARs dose constraint and the prescribed dose for the target (HR-CTV) recommended by the GEC-ESTRO, and the American Brachytherapy Society (ABS), all the treatment plans were created using the Oncentra treatment planning (Elekta, Stockholm, Sweden) system and based on TG 43 algorithm. The HRCTV was given an 80–90Gy EQD2 (biologically equivalent dose in 2-Gy fractions) prescription dose, assuming α/β = 10. The bladder was given a maximum D_2cc_ of 90Gy EQD2, and the rectum was given a maximum D_2cc_ of 75 Gy EQD2, assuming α/β = 3. The prescription dose in HDR brachytherapy ranges from 4-6 Gy/fraction (**Table 1**). In the treatment planning, we use a graphical optimization approach to repeatedly optimize the plan until the dose administered to 90% of the HRCTV reached the prescription dose. The dose distribution in each plan was scrutinized by physicians and physicists to ensure the dose distribution was clinically acceptable before treatment.

### 2.3 DVH prediction modeling

#### 2.3.1 Feature reduction

**Figure 2:**
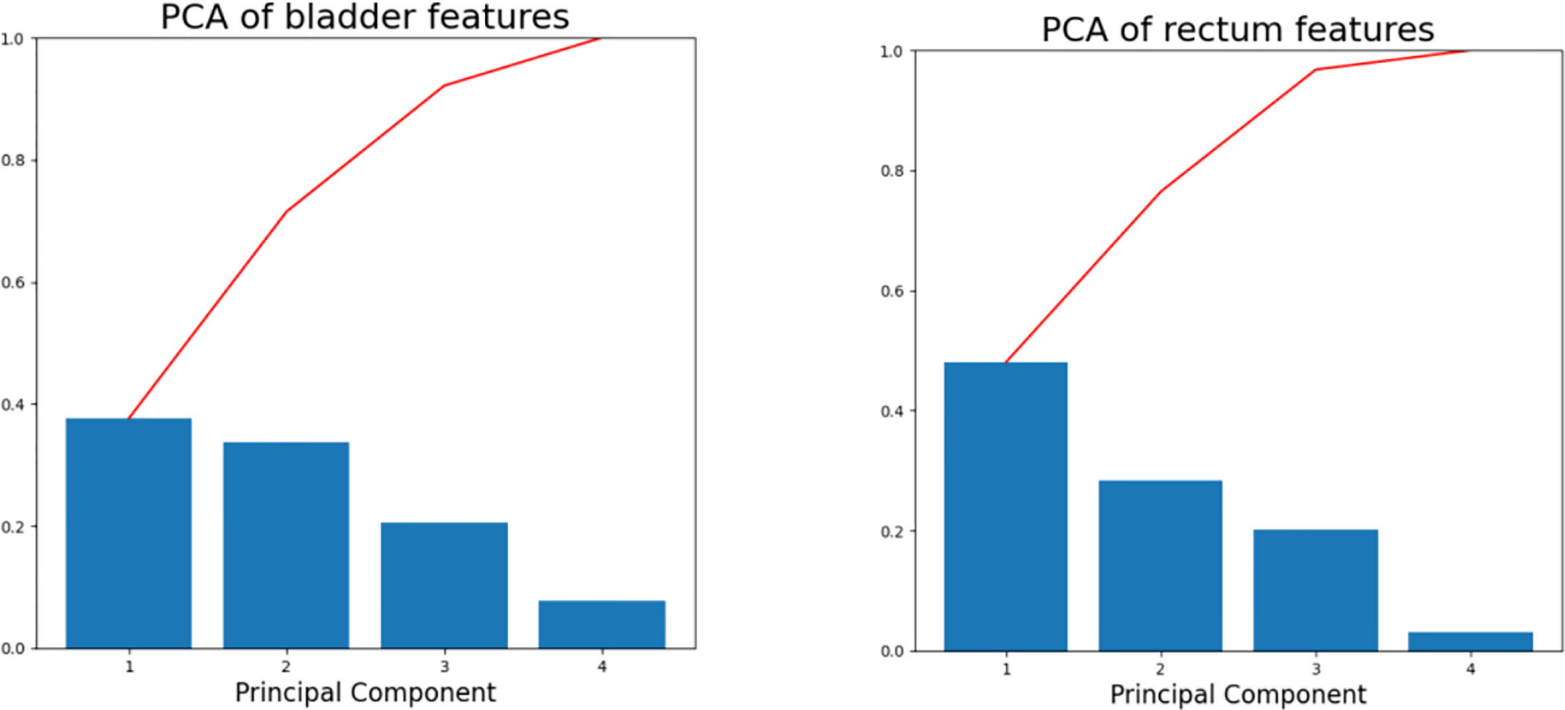
Scree plots of the bladder (left) and rectum (right) after PCA was applied to the training database. The percentage of variance represented by individual PC is plotted in descending order as blue bars, and the cumulative total is represented by red lines.

Principal component analysis (PCA) has been applied to the brachytherapy plans to reduce the data dimensions and the model complexity (18, 27). PCA enables the transformation of a large set of variables into a smaller one containing most of the information. In this study, four features closely related to the dose distribution were processed using PCA to further reduce their dimensions: HRCTV volume, the distance between the centroids of the D_2cc_ and the HRCTV, prescription dose, and the average distance from D_2cc_ to the margin of the HRCTV. PCA simplifies the diversity of the dose distribution into a few principal component directions, and the individual variations can be represented by a small number of principal components. In our study, the first three principal components account for more than 90% of the variance (**Figure 2**) in both bladder and rectum, and thus were employed in the subsequent kNN similarity matching process for case selection.

#### 2.3.2 k-Nearest similarity matching

To take full advantage of the proposed feature, we incorporated k-Nearest Neighbors (kNN) similarity matching (28), a known robust non-parametric regression method to reduce model complexity. The purpose of kNN is to select a subset of training cases that matches closest to the validation case to create a new training dataset for subsequent model training. After recasting the training dataset along the principal component axes, the Euclidean distance between the single case in the validation set and each case in the training dataset was calculated separately in three principal component dimensions. kNN was thus used to retrieve k cases having the smallest Euclidean distance (bladder: k=30, rectum: k=20) in the training dataset.

#### 2.3.3 Kernel density estimation

**Figure 3:**
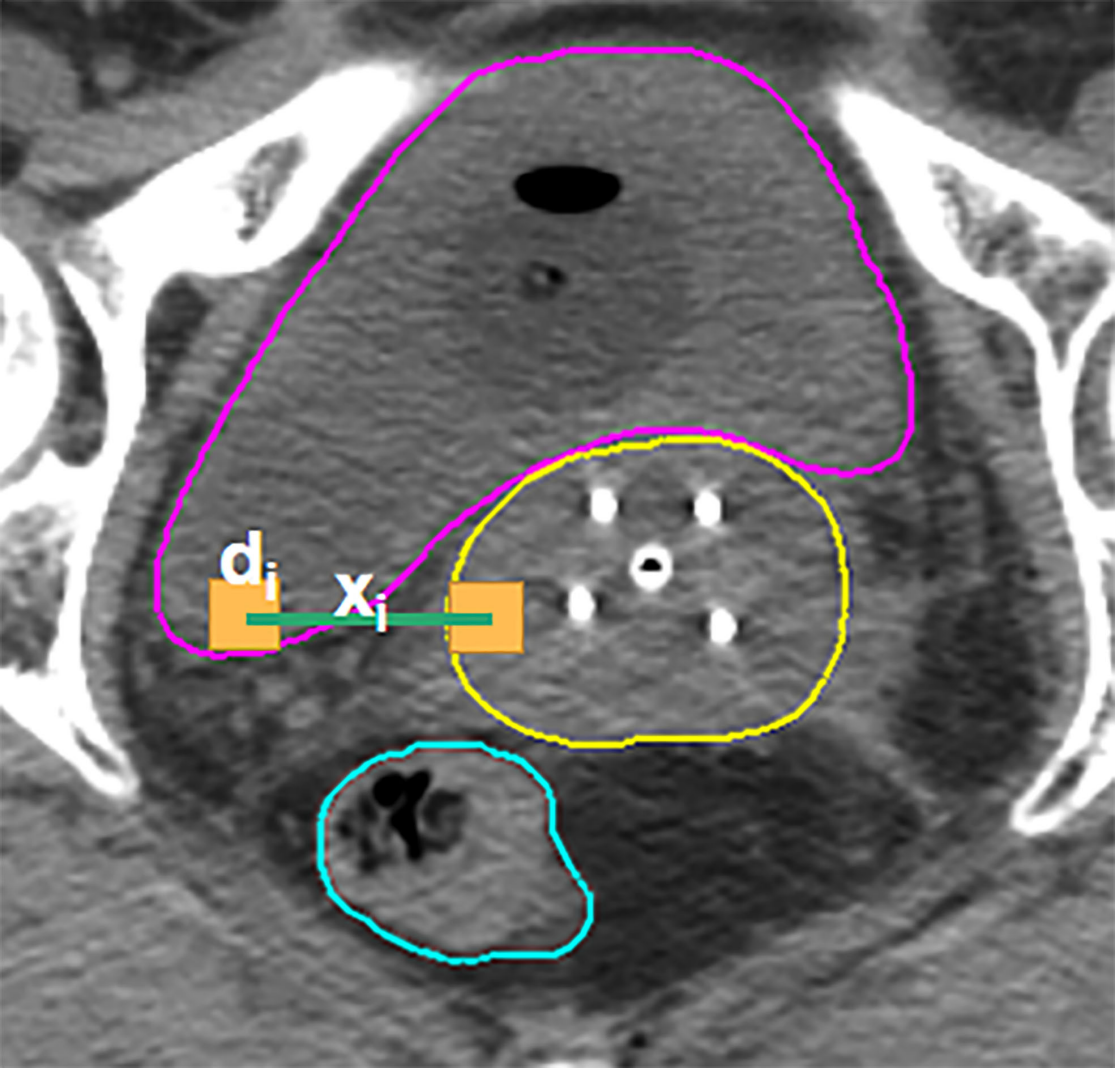
Illustration of the KDE method in the brachytherapy treatment planning. For each voxel in the OAR, xi is the distance between this voxel and its closest voxel on the HRCTV surface and di is the dose received in each voxel inside the OAR.

Considering that the determinant factor for dose levels near the HRCTV is the distance to the HRCTV, the histogram of distances from voxels in OARs to the HRCTV surface, which is the distance-to-target histogram (DTH), is a natural choice for predictive features. Thus, we implemented kernel density estimation (29, 30) for model training in this study. In KDE, for each voxel inside the OAR, we measure the closest distance from the voxel to the HRCTV boundary, and the distance was denoted by x_i_ = x_1_, x_2_,… x_i_ (**Figure 3**). The second step was to estimate the joint probability P_D, xi_ (d, xi) of the dose d and the distance x, in which d was the corresponding dose in each voxel inside the OAR. Thirdly, we estimated the distance probability distribution P_Xi_ (xi), and calculated the conditional probability through: P_D|xi_ (d|xi) = P_D, Xi_ (d, xi)/P_Xi_(xi)

In the prediction part, we estimated the new distance distribution probability $P_{xi}^{\#}(\text{xi)}$ for each case from the validation set. Based on the calculated conditional probability in the training process, the prediction of the dose distribution for a new patient in the validation set can be calculated as: $P_{D}^{\#}(d)=\sum_{i}{\text{P}}_{\text{D}|\text{Xi}}(\text{d}|\text{xi})\;\cdot \;P_{xi}^{\#}(\text{xi})$ . In the final step, DVH was defined as a function of the dose d and the probability that a random variable D was larger than or equal to d: $\text{DVH(d})\;=\;1-\text{P(D}\leq \text{d})\;=\;1-\;\int_{0}^{\text{d}}{\text{P}}_{\text{D}}^{\#}\text{(s})\text{ds}$ .

### 2.4 Model validation

To quantitatively measure the prediction accuracy of the proposed model, we set the actual clinical plan DVHs as a baseline for comparison. Specific dose-volume indices including D_2cc_, D_1cc_, D_0.1cc_, D_max_, and D_mean_, were extracted and analyzed, where D_xcc_ represented the minimum dose received by x cm^3^ of an OAR. Absolute residuals of predicted value and the actual value (|ΔD_2cc_|, |ΔD_1cc_|, |ΔD_0.1cc_|, |ΔD_max_|, and |ΔD_mean_|) were calculated to assess the level of agreement between the predicted DVHs and the actual plan DVHs. Standard deviation (σ) over the residuals was considered a measure of model error. The mean squared error (MSE) was calculated for all planned and predicted D_2cc_ values.

### 2.5 Statistical analysis

Significant differences were determined using a two-sided paired t-test. Correlations between predicted parameters and actual parameters were tested by performing the Pearson correlation test. Kruskal-Wallis ANOVA was employed to identify differences among various applicators. All statistical data analyses were performed using Python.

## 3 Results

**Figure 4:**
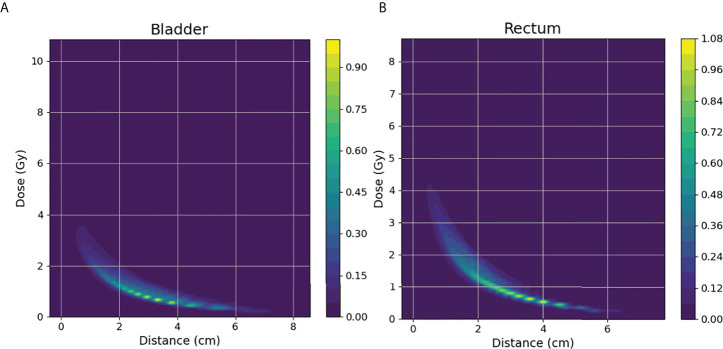
**(A, B)** depict the KDE model for bladder(left) and rectum(right) of 170 training cases.

**Figure 5:**
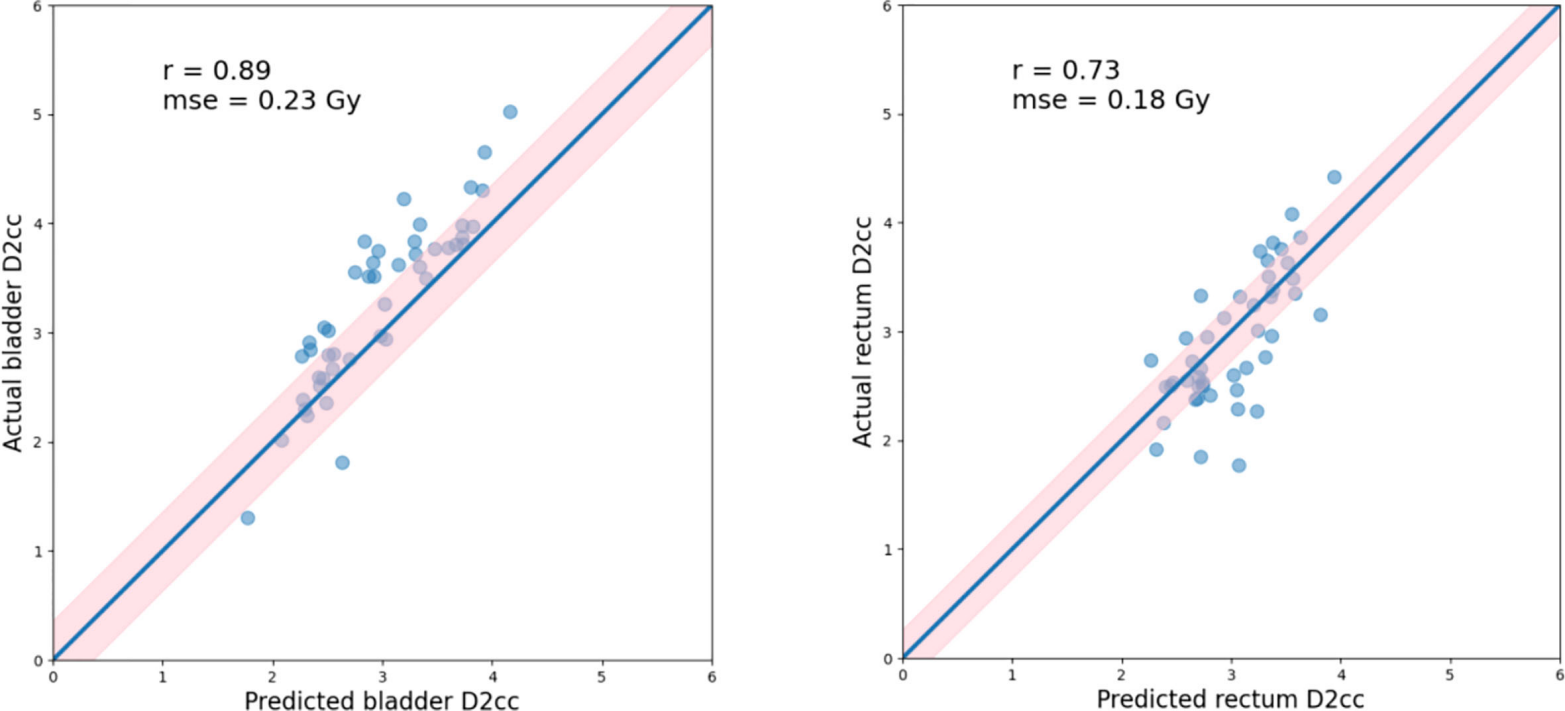
Actual vs. predicted D2cc for the bladder(left) and rectum(right), as well as the Pearson correlation coefficients (r), standard deviation (indicated by σ as well as light pink shaded area). Blue lines indicate the theoretically ideal predictions.

For each test case, we built a model trained with the selected k cases (bladder: k=30, rectum: k=20) from a total of 170 training cases. The model accuracy was evaluated using a separate validation dataset consisting of 46 cases. The kernel density model based on 170 cases is shown in **Figure 4**. There was no statistically significant difference between the predicted and actual D_2cc_ values for the bladder (p = 0.74), rectum (p = 0.57) in the validation group. As shown in **Table 2**, the MSE value of D_2cc_ for the bladder was 0.23, and for rectum was 0.18. In bladder, the absolute difference between predicted plans and actual plans in D_2cc_, D_1cc_, D_0.1cc_, D_max_ and D_mean_, were 0.38 ± 0.29, 0.4 ± 0.32, 0.43 ± 0.36, 0.97 ± 0.66 and 0.13 ± 0.09, respectively. For the corresponding rectum, the absolute difference values were 0.34 ± 0.27, 0.38 ± 0.33, 0.63 ± 0.57, 1.41 ± 0.99 and 0.23 ± 0.17, respectively. The model prediction error in D_2cc_ to the bladder, and rectum was within 0.3 Gy, as quantified by the standard deviation (**Figure 5**).

**Table 2:** Model performances for bladder and rectum.

	MSE (D2cc)	|ΔD2cc|	|ΔD1cc|	|ΔD0.1cc|	|ΔDmax|	|ΔDmean|
Bladder	0.23	0.38 ± 0.29	0.4 ± 0.32	0.43 ± 0.36	0.97 ± 0.66	0.13 ± 0.09
Rectum	0.18	0.34 ± 0.27	0.38 ± 0.33	0.63 ± 0.57	1.41 ± 0.99	0.23 ± 0.17

The mean squared error and the absolute difference between predicted values and actual values were calculated.

**Figure 6:**
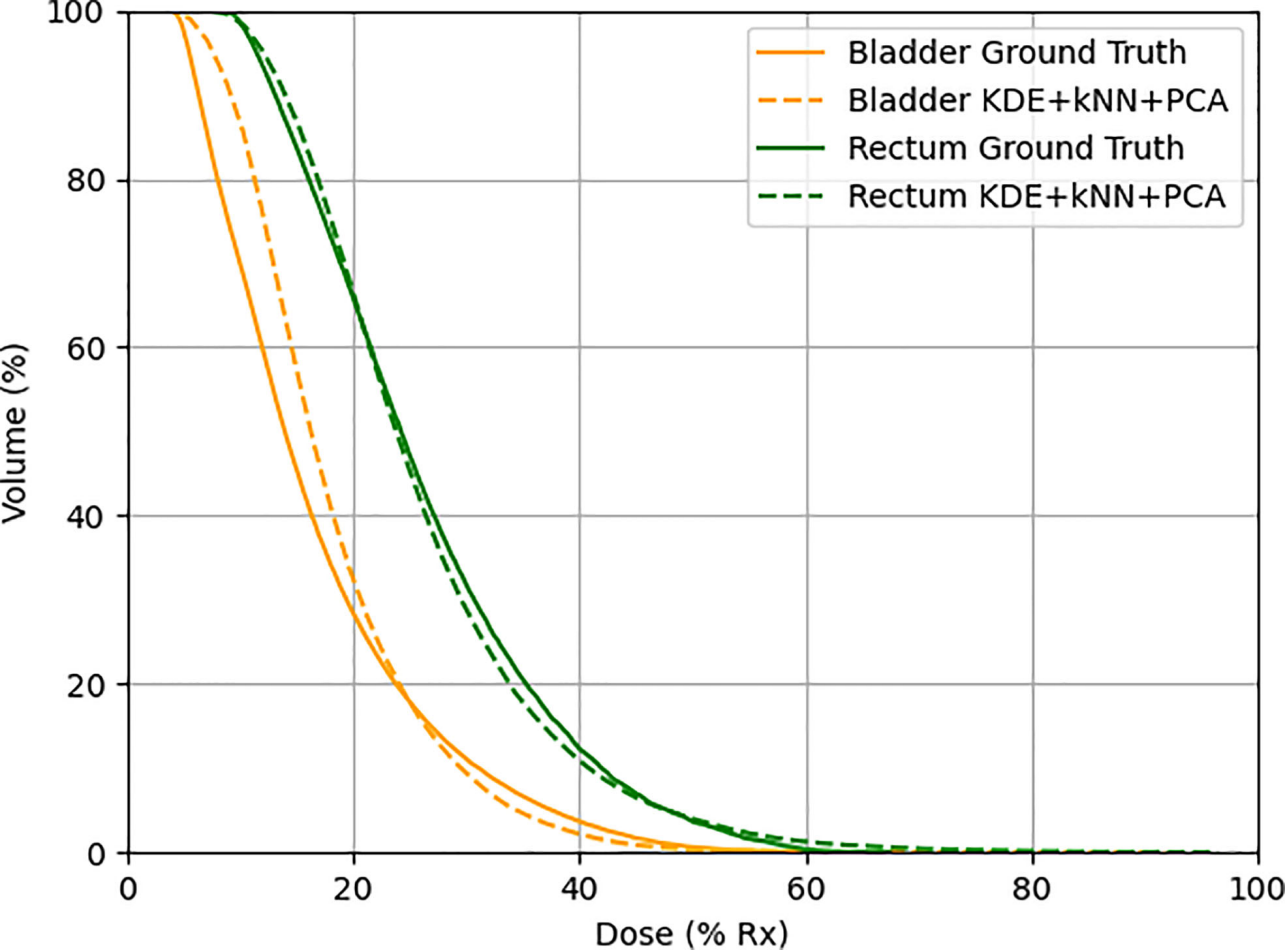
One example in the validation set demonstrating actual (solid line) and predicted (dotted line) DVHs. Orange lines represent the bladder, and green lines represent the rectum.

The model-predicted DVHs were compared to their corresponding DVHs in the actual plans to assess the model’s prediction accuracy. The DVHs of one example in the validation set were plotted and compared, as shown in **Figure 6**.

## 4 Discussion

Knowledge-based planning in EBRT has demonstrated its effectiveness across multiple disease sites. However, it is still less investigated in high-dose-rate brachytherapy. This study developed a KBP method for DVH prediction in HDR-BT. Theoretically, such a KBP model can be trained using gold-standard datasets and serve as quality assurance tools in the clinic to identify suboptimal plans in treatment planning prospectively. Therefore, this KBP method has a great potential to assess the treatment plan quality and offer guidance for following plan optimization.

Different from previous studies, in which only T+O applicator was investigated (25, 26, 31), we involved different applicator sets including T+O, Vaginal Multi-Channel applicator, Ovoid applicator, free needles, 3D printed applicators, and T+N applicators. Overall, our results show slightly better accuracy in D_2cc_ than Yusufaly et al. (25): bladder σ was 0.36 Gy vs. 0.46 Gy, rectum σ was 0.42 Gy vs. 0.47 Gy. (Since Yusufaly used relative error, here we also used the results of relative error for comparison.) To evaluate whether the applicator types would affect the predictive accuracy, we applied the Kruskal-Wallis analysis of variance (ANOVA) test in the validation dataset. The ANOVA revealed that D_2cc_ has no significant statistical difference among different applicators (p=0.109). A possible explanation for this might be that the combination of kNN and PCA selected similar training cases for each validation case, reducing the variation in dose distribution caused by different applicators.

kNN was used to select k plans in the training dataset that mostly resemble the case in the validation dataset for model training. Once we determined the k value, we would neglect or hardly consider the information of other nearest neighbors. Thus, reducing the number of cases for training naturally comes at the expense of accuracy. The benefit of the case reduction is that smaller data set is easier to explore and analyze. It eliminates redundant and irrelevant variables and gives rise to an easier and faster training process in machine learning. A proper k value is crucial for model training and an inappropriate k value may yield unstable performance. To select the most appropriate k value, we run the kNN algorithm on 20 test cases with k values ranging from 10-40. The k value achieved the best model performance in D_2cc_ was used in model training (**Table 3**). Thus, we select k=30 for bladder and 20 for the rectum.

**Table 3:** Mean square error of predicted D_2cc_ using different k values.

k	Bladder	Rectum
10	0.283	0.094
20	0.215	0.091
30	0.210	0.106
40	0.215	0.110

**Figure 7:**
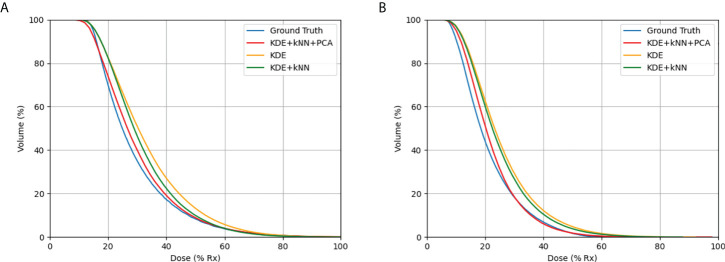
Comparison of DVHs in actual plan and the predicted DVHs using different methods: **(A)** bladder, **(B)** rectum. The method using KDE, kNN, and PCA produced the most accurate results when compared to actual DVHs.

PCA was combined with kNN for better similarity matching in case selection. The main function of PCA was to reduce feature dimensionality in an interpretable way, while preserving as much information as possible at the same time. In our study, the first three principal components were used for kNN similarity matching. To verify the effectiveness of the PCA and kNN in model performance, we tested two different methods, KDE only, and KDE combined with kNN and PCA, separately. As shown in **Table 4**, results showed that the all-inclusive approach which combined KDE, kNN and PCA achieved the best D_2cc_ performance for both bladder and rectum. The all-inclusive method showed a significantly better prediction performance than KDE only, with an improvement of 30.3% for bladder and 33.3% for rectum. **Figure 7** depicts one example predicted DVHs and actual DVHs (ground truth) for bladder and rectum. The DVHs predicted using all-inclusive method had the lowest difference from the actual DVHs.

**Table 4:** Comparison of model performances using different methods.

		MSE (D2cc)	|ΔD2cc|	|ΔD1cc|	|ΔD0.1cc|	|ΔDmax|	|ΔDmean|
**Bladder**	KDE	0.27	0.44 ± 0.28	0.47 ± 0.29	0.5 ± 0.42	2.1 ± 1.9	0.32 ± 0.2
	KDE+kNN+PCA	0.23	0.38 ± 0.29	0.4 ± 0.32	0.43 ± 0.36	0.97 ± 0.66	0.13 ± 0.09
**Rectum**	KDE	0.27	0.43 ± 0.31	0.45 ± 0.34	0.54 ± 0.49	0.93 ± 0.72	0.31 ± 0.23
	KDE+kNN+PCA	0.18	0.34 ± 0.27	0.38 ± 0.33	0.63 ± 0.57	1.41 ± 0.99	0.23 ± 0.17

The mean squared error and the absolute residual between predicted values and ground truth were calculated.

DVH prediction modeling is often a complex, not a simple problem. There are still several sources of error limiting the model"s ability to predict a satisfactory DVH. In this investigation, the main error came from the nonconformity of the HRCTV dose distribution, and 100% prescription dose line (contour of V_100%_). In KDE, since we do not have the contour of 100% prescription dose line for validation cases, we use HRCTV instead. We calculated the probability distribution based on the assumption that HRCTV achieved exactly 100% prescription dose. However, the dose distribution inside the HRCTV was determined by the source dwell position and dwell time, and the dose distribution in brachytherapy are highly constrained around applicators. The HRCTV and 100% prescription dose line can be slightly inconsistent if the case has inadequate or inappropriate applicator positions in some slices, especially in the slices at the beginning or the end of HRCTV. That is to say, the bias between the HRCTV contour and the 100% prescription dose line would cause error in the subsequent dose probability estimation. Thus, we applied a contour correction method to adjust the HRCTV contour.


$$\text{Mean }=\;\frac{1}{\text{n}}\sum^{}\frac{1}{{\text{r}}_{\text{i},\text{j}}^{2}}$$

where i = 1, 2, 3…n, n is the number of points in the HRCTV boundary, j = 1, 2, 3… m, m is the number of applicators, r_i,j_ represents the distance between the point in the HRCTV boundary to the applicator.

**Figure 8:**
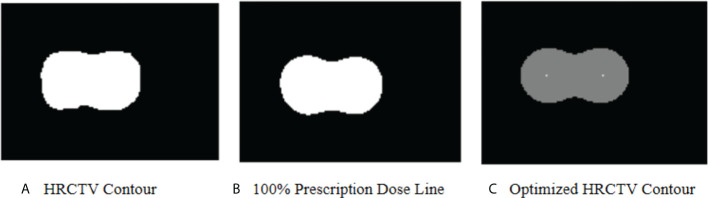
**(A)** shows the HRCTV contoured by a radiation oncologist in an actual plan. **(B)** shows the 100% prescription dose line in an actual plan. **(C)** shows the optimized HRCTV contour based on the correction method. In KDE method, we assume **(A)** matches with **(B)** perfectly. In this case, the optimized HRCTV has a better similarity with the 100% prescription dose line.

**Figure 8** shows the optimized HRCTV contour based on the correction method. The Dice Similarity Coefficient (DSC) value was used to evaluate the matching degree after correction. The DSC values between the optimized contour and 100% prescription dose line indicate a slightly better match degree than the original HRCTV contour, but ultimately these corrections made unstable and modest improvements ranging from -0.023-0.057 in D_2cc_ prediction accuracy. Thus, we did not integrate this correction into our machine learning algorithm. This limitation will become the direction of our future research.

To sum up, this work presents a KBP method to predict DVH for OARs in brachytherapy treatment. Patient anatomical features in previously treated cases were learned to predict DVHs for new patients. The predictions based on the individual patient geometry could motivate planners to go beyond the dosimetric constraints imposed by protocols to improve planning and provide better dose sparing for OARs. In our study, the entire process, including case selection, model construction, and DVH prediction, can be completed within one minute, which is acceptable in clinical application. Initial results have shown great potential in making this KBP model a quality control tool in treatment planning. Future studies will focus on the feasibility verification of using this model as a quality control tool in clinical practice. Another research direction is to re-plan those plans with D_2cc_ exceeding the prediction interval to improve plan quality and facilitate customized treatment planning for each patient.

## 5 Conclusion

In this paper, we proposed a machine learning method based on KDE, kNN, and PCA to predict the DVH in HDR-BT. The DVH for a new treatment plan was estimated using patient-specific anatomical information and an estimation model trained from prior plans. To our knowledge, this is the first KBP method that can predict the DVHs in patient who was treated with interstitial applicators, intracavitary applicators or both. The preliminary results have verified the model’s effectiveness in OAR dose estimation and its potential in providing guidance for brachytherapy planning in the future.

## Data availability statement

The original contributions presented in the study are included in the article. Further inquiries can be directed to the corresponding author.

## Ethics statement

The studies involving human participants were reviewed and approved by Shanghai Sixth People’s Hospital. Written informed consent for participation was not required for this study in accordance with the national legislation and the institutional requirements.

## Author contributions

ZL and KC : data acquisition, data processing, statistics, and writing; ZY, QZ, XY, ZL and JF: reviewing and editing. All authors contributed to the article and approved the submitted version.

## Funding

This research was supported by the Science and Technology Project of Shanghai Municipal Science and Technology Commission (No.22Y31900500).

## Conflict of interest

The authors declare that the research was conducted in the absence of any commercial or financial relationships that could be construed as a potential conflict of interest.

## Publisher’s note

All claims expressed in this article are solely those of the authors and do not necessarily represent those of their affiliated organizations, or those of the publisher, the editors and the reviewers. Any product that may be evaluated in this article, or claim that may be made by its manufacturer, is not guaranteed or endorsed by the publisher.
